# A Cluster-Based 3D Reconstruction System for Large-Scale Scenes

**DOI:** 10.3390/s23052377

**Published:** 2023-02-21

**Authors:** Yao Li, Yue Qi, Chen Wang, Yongtang Bao

**Affiliations:** 1 State Key Laboratory of Virtual Reality Technology and Systems, Beihang University, Beijing 100191, China; 2 Peng Cheng Laboratory, Shenzhen 518055, China; 3 Qingdao Research Institute of Beihang University, Qingdao 266104, China; 4 School of Computer Science and Engineering, Beijing Technology and Business University, Beijing 100048, China; 5 College of Computer Science and Engineering, Shandong University of Science and Technology, Qingdao 266590, China

**Keywords:** large-scale scene, structure from motion, multi-view stereo, mesh optimization, cluster system, large-scale 3D-reconstruction system

## Abstract

The reconstruction of realistic large-scale 3D scene models using aerial images or videos has significant applications in smart cities, surveying and mapping, the military and other fields. In the current state-of-the-art 3D-reconstruction pipeline, the massive scale of the scene and the enormous amount of input data are still considerable obstacles to the rapid reconstruction of large-scale 3D scene models. In this paper, we develop a professional system for large-scale 3D reconstruction. First, in the sparse point-cloud reconstruction stage, the computed matching relationships are used as the initial camera graph and divided into multiple subgraphs by a clustering algorithm. Multiple computational nodes execute the local structure-from-motion (SFM) technique, and local cameras are registered. Global camera alignment is achieved by integrating and optimizing all local camera poses. Second, in the dense point-cloud reconstruction stage, the adjacency information is decoupled from the pixel level by red-and-black checkerboard grid sampling. The optimal depth value is obtained using normalized cross-correlation (NCC). Additionally, during the mesh-reconstruction stage, feature-preserving mesh simplification, Laplace mesh-smoothing and mesh-detail-recovery methods are used to improve the quality of the mesh model. Finally, the above algorithms are integrated into our large-scale 3D-reconstruction system. Experiments show that the system can effectively improve the reconstruction speed of large-scale 3D scenes.

## 1. Introduction

As large-scale 3D models are the basis for smart cities, there has always been an urgent need for digitizing large-scale scenes. There are a number of applications and research fields that can benefit from 3D models of large-scale scenes, including digital cities, virtual reality, augmented reality and digital twins. However, rapidly reconstructing high-quality, large-scale 3D scene data remains a difficult task in computer vision and graphics research. First, as the scale of the scene increases, the number of input images increases significantly.

When using a single computational node, 3D reconstruction will exceed its own computational and storage capacity, leading to the failure of large-scale-scene 3D-reconstruction tasks. Additionally, in large-scale 3D reconstruction, it is assumed that even if a single computing unit could complete the reconstruction task, the vast amount of data would often lead to a longer computing time.

Finally, the scale of the 3D data of the reconstructed large-scale scene needs to be increased, thereby, resulting in a high degree of data redundancy and a significant number of noise problems, which is not conducive to the expression and rendering of 3D data. To address the problem of large-scale 3D-scene reconstruction that a single computing node cannot handle, we propose a cluster-based camera-graph-structure clustering algorithm and a cluster-based global camera pose-registration algorithm. All camera positions are converted into graph-structured nodes, and several strongly correlated camera subsets are constructed using graph-structured partitioning.

A reasonable subset of the parallel computational data is assigned to each computational node in the cluster. The overlapping camera information among the subgraphs is used to optimize and complete the task of registering global camera positions. In conclusion, our proposed algorithm relies on a divide-and-conquer framework to break through the limitations of a single computational node and uses a cluster approach to handle the 3D-reconstruction task of large-scale scenes. To maximize the utilization of computational resources on a single computational node, we propose a GPU parallel depth-estimation algorithm based on patch matching in the dense point-cloud reconstruction phase to assess the candidate solutions’ merit using an improved normalized correlation score.

During the iterative process, the search neighborhood size is continuously adjusted according to the distribution location of the solutions to accelerate the convergence speed further. This reduces the time needed to solve the dense reconstruction problem on a single computational node and alleviates the time-consuming problem of depth-map estimation when reconstructing dense point clouds of large-scale 3D scenes. The 3D reconstructed mesh has disadvantages, including data redundancy, a non-uniform structure, spatial noise and non-fine detail features. We propose a 3D mesh-optimization method based on cluster geometric detail recovery.

First, we simplify the mesh to maintain the detailed features of the model while reducing the redundancy of the mesh model as much as possible. Secondly, in the process of mesh smoothing, we propose a mesh-smoothing homogenization algorithm based on the second-order umbrella operator, which makes the triangular surface slices regular and uniform, thus, reducing the noise and anomalies of the mesh. Finally, we construct an energy function using the image color-consistency term and the Laplacian smoothness term, which not only removes noise and outliers but also restores the detailed features of the mesh.

Based on the above method, our developed large-scale 3D modeling system, BHReconstruction, realizes the need for cluster-based, high-efficiency reconstruction of large-scale 3D scenes. BHReconstruction supports exporting mainstream 3D model formats and the real-time rendering of 3D models, thereby, meeting the needs of further applications, such as surveying and mapping, 3D maps, digital cities, digital cultural relics, virtual reality and augmented reality. BHReconstruction consists of three modules: the reconstruction configuration module, 3D-reconstruction module and rendering module.

Our system provides two modes for different users: (1) simple mode, which only needs to configure the basic options to achieve one-click reconstruction of large-scale 3D scenes and (2) expert mode, which provides detailed parameter settings to meet the specialized 3D-reconstruction needs of professionals who have mastered 3D-reconstruction theory.

In summary, this study makes the following contributions to the existing literature:

- We propose a cluster-based method for clustering the camera graph algorithm. A divide-and-conquer framework is used to precisely partition the camera graph into several subsets. The algorithm ensures weak correlations between subsets and strong correlations within subsets, which allows the subsets to perform in parallel local camera pose-estimation tasks on cluster nodes.
- We propose a cluster-based global camera pose-alignment algorithm. Using the overlapping camera positions between each subgraph for global camera pose fusion, we mainly solve the nonlinear optimization problem of rotation and translation in the camera pose to obtain a more accurate global camera pose.
- We propose a GPU parallel fast depth-estimation method based on patch matching. The candidate solutions are measured by an improved normalized correlation score, which makes the parallel estimation of image depth values more efficient.
- We propose a cluster-based mesh optimization for the geometric detail-recovery method, which uses the proposed second-order umbrella operator to enhance the mesh’s uniformity and increase the mesh model’s fidelity.
- Compared with similar open-source libraries and commercial reconstruction software, our system can reconstruct large-scale city-level 3D scenes in a cluster environment with one click and has a faster 3D-reconstruction speed within a certain reconstruction quality.

## 2. Related Work

### 2.1. 3D Reconstruction Methods

#### 2.1.1. Structure from Motion

In the sparse point-cloud reconstruction stage, Structure from Motion (SFM) is the core algorithm with the primary goal of recovering the internal and external parameters of the camera. SFM is mainly classified into incremental SFM, global SFM and hybrid SFM methods [1,2,3]. Incremental SFM [4,5,6,7] constantly uses bundle adjustment [8,9,10] to correct camera pose and sparse point clouds. Kneip et al. [11] proposed total SFM reconstruction using the P3P algorithm and RANSAC to reject outliers and reduce drift errors. Since bundle adjustment is the most time-consuming part of incremental SFM, Wu et al. [12] proposed a GPU-based bundle adjustment that reduces the bundle adjustment time. Eriksson et al. [13] used a distributed approach to chunk the camera pose, and both methods were able to perform the bundle adjustment task in a highly parallel manner.

Compared to incremental SFM, which performs bundle adjustment multiple times, global SFM [14,15,16,17,18,19,20,21,22,23,24] performs only one bundle adjustment after computing all camera poses. In order to calculate the global rotation matrix of all cameras, the global SFM algorithm first calculates the global rotation matrix. Afterward, it calculates the global translation vector for all cameras, and finally it determines the global position of the camera center based on global rotation and translation. Crandall et al. [25] developed a method to reject mismatches using Markov random fields.

Cui et al. [26] introduced auxiliary information to speed up the sparse point-cloud reconstruction process. Sweeney et al. [27] proposed using an optimized camera map to accelerate the global SFM process. The key to global SFM is the computational work performed to determine the global camera poses. Sweeney et al. [24] proposed an optimization method based on the Hessian matrix, with the time and space complexity of cubic and square levels determined for the number of input images.

Hybrid SFM [28] combines the advantages of incremental SFM and global SFM. Zhu et al. [29] proposed a camera map node-clustering algorithm to generate overlapping camera clusters. This algorithm performs local incremental SFM reconstruction of relative camera poses applied to the global pose-averaging framework [30]. However, this method can lose camera and feature-point correspondences. Zhu et al. [31] divided all images into multiple partitions, which allow for parallel local camera pose computation because these partitions retain strong data associations. This distributed framework significantly improves the efficiency and robustness of extensive scene reconstruction.

#### 2.1.2. Multi-View Stereo

Multi-view stereo (MVS) algorithms [32,33,34,35] can be classified into four categories at the dense point-cloud reconstruction stage: feature-point-based MVS, voxel-based MVS, depth-map merging-based MVS and deep-learning-based MVS. Feature-point-based MVS [36,37,38] uses different point-cloud evolution strategies for dense reconstruction and is limited by its incremental, iterative nature and the difficulty of parallelizing it. Habbecke et al. [39] reconstructed sparse point clouds and then extracted dense points from the quasi-dense parallax maps they constructed, using this method to overcome the feature-point sparsity problem.

Goesele [40] proposed an MVS method that utilizes local and global image information. Furukawa [41] built on these previous methods by proposing a faceted slice-based MVS method that starts from the feature points of a sparse point cloud and iteratively extends iterations to remove false matches according to photometric and geometric visibility constraints.

Voxel-based MVS [42,43,44] discretizes the 3D space into a regular cubic grid and determines whether each voxel lies on the surface of a 3D object to represent the 3D object. Seitz [42] proposed a voxel coloring-based method to estimate the surface, searching the entire 3D space through the depth traversal to identify blocks of voxels with specific colors. Vogiatzis [43] used the graph cut optimal algorithm to estimate the minor surface containing the maximum volume, which is limited by the voxel discretization resolution.

The depth-map merging-based MVS [45,46,47,48,49] uses depth images to represent 3D scenes, allowing the processing to be parallelized and concentrated on one reference image and a few neighboring images in one operation. By relieving the computational load on a single computational node, large-scale reconstruction of 3D scenes can be performed. Schonberger [50] applied a patch-window-matching algorithm based on normalized cross-correlation (NCC) scores to generate depth maps. Merrell [51] used a computationally less expensive algorithm to quickly generate depth maps with considerable noise and overlapping regions and to obtain an overall 3D point cloud with visibility constraints.

Researchers have recently started experimenting with deep-learning methods to solve the MVS depth-estimation problem. MVSNet [52] first used an end-to-end neural network to impute the depth map, which somewhat solved the scene size limitation. The network transformed the neighborhood image under a single-strain transformation to the reference view, constructed the cost cube and later applied a 3D UNet to impute the depth values. Yu [53] proposed Fast-MVSNet, which relies on sparse cost cubes and Gaussian Newton layers to improve the speed of MVSNet.

All the above networks used the DTU [54] dataset for training. However, in order to be able to apply the network to practical applications, some other researchers have attempted to construct loss functions using projection consistency errors to train the network in an unsupervised way, such as Unsupervised MVSNet [55] and MVS2 [56]. Some networks add semantic information and data augmentation processes to enhance the accuracy, such as M3VSNet [57] and JDACS-MS [58].

#### 2.1.3. Mesh Optimization

Researchers have studied ways to simplify 3D mesh models for large-scale scenes, such as overcoming problems associated with data-volume redundancy. Garland et al. [59] proposed a mesh-simplification method based on a quadratic error metric to obtain a simplified 3D mesh model. This method was based on constructing a quadratic error matrix to represent the errors of vertices and surfaces. Hoppe et al. [60] improved the above method by optimizing the storage, computational efficiency and model quality.

Williams et al. [61] developed a perceptual model for edge-folding operations considering texture-coordinate deviation, illumination contrast and dynamic illumination. Lindstrom et al. [62] proposed an image-driven 3D mesh-simplification method that uses texture images to calculate the cost of edge folding.

Wang et al. [63] proposed a 3D mesh-simplification method based on curvature variation by introducing the average curvature of vertices and the curvature variation of the triangular surface piece where the vertex is located as the folding edge cost. An et al. [64] presented a 3D mesh-simplification method based on multi-view image saliency, which considers the vertices’ color information and detail features to ensure the minimum loss of vertex saliency after each simplification operation. Jiang et al. [65] introduced the distance error as an additional metric, examining the effects of both curvature and distance error on the detail retention of simplified models.

In the 3D-reconstruction process, there are inevitably various noises and perturbations in the mesh model, and many narrow triangular facets may exist. Therefore, it is necessary to smooth the mesh to eliminate mesh surface noise and improve the quality of the triangular facets while maintaining the triangular mesh characteristics. Taubin [66] proposed a weighted Laplacian smoothing method by adding a weighting factor to the Laplacian operator, which controls the deformation of the model to an extent. Desbrun [67] proposed the mean curvature method by adding a weighting factor along the vertices of the surface.

Fleishman [68] extended bilateral filtering from 2D images to triangular meshes with positive results. Bajbaj [69] improved the smoothness using the bilateral filtering technique. Hildebrandt [70] not only preserved the detailed feature edges of the model but also preserved and recovered the nonlinear surface features of the model by specifying the mean curvature. Kai [71] applied bilateral filtering to the surface normals and achieved smoothness while maintaining the detailed features of the model. However, dual filtration still caused the model to experience shrinkage effects.

### 2.2. 3D Reconstruction Libraries and Software

Currently, several open-source libraries and systems are capable of completing 3D reconstructions of parts of pipelines or entire pipelines. VisualSFM [72] is a GUI application that implements the SFM algorithm for sparse point-cloud reconstruction. Using the output data of this program with libraries, such as MVE and openMVS, subsequent 3D-reconstruction tasks can be completed. Furukama proposed the patch-based MVS (PMVS) [41] algorithm and created the open-source PMVS library, which uses the sparse reconstruction results to diffuse them around the space and produce a directed point cloud through three steps: matching, expansion and filtering.

Clustering views for MVS (CMVS) [73] is an improved version of PMVS that clusters images to reduce the amount of data. MVE [74] is an open-source library for the end-to-end pipeline implementation of image-based geometric reconstruction. It has SFM, MVS and surface reconstruction capabilities. Since MVE lacks texture-reconstruction capabilities, it can be used in conjunction with MVS-Texturing [75], the first comprehensive texture framework for large-scale, real-world 3D reconstruction as proposed by Waechter.

OpenMVG [76] is able to conduct the entire sparse point-cloud reconstruction phase, from feature detection and matching to recovering structures from motion. OpenMVS [77] is another well-known open-source library for 3D reconstruction that works perfectly with OpenMVG. Its main features are dense point-cloud reconstruction, surface reconstruction, surface refinement and texture mapping. OpenMVS inputs are sparse point clouds and camera positions, and its outputs are 3D meshes with textures. Colmap [78] is a general-purpose SFM with graphical and command-line interfaces and an MVS pipeline, whose MVS part needs to be implemented based on CUDA.

In addition to 3D-reconstruction capabilities, many commercial 3D-reconstruction software packages include photogrammetry capabilities for professional use. Photoscan [79] is software that automatically generates high-quality 3D models based on images. Pix4Dmapper is standard 3D-reconstruction and aerial-photogrammetry software that includes point-cloud reconstruction, 3D-mesh reconstruction, elevation-map generation and other functions. In addition, Pix4Dmapper developed mobile applications that allow the use of drones as mapping tools.

These applications allow control over of the drone’s flight path to ensure sufficient overlap between images for photogrammetric processing. ContextCapture is powerful, professional 3D realistic modeling software with clustering capabilities that enables multiple machines to collaborate and process data in parallel. The above software systems have robust 3D reconstruction, tilt photography, measurement, and mapping functions. However, the software’s operation is relatively complex and more suitable for professional use, and the software is expensive.

## 3. System Design

### 3.1. Overall Structure

**Figure 1:**
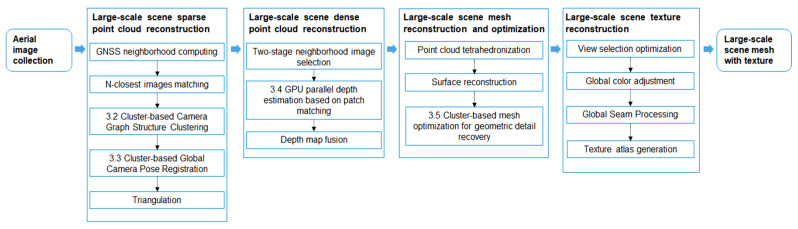
Pipeline of the proposed system.

To solve the problems in large-scale scene 3D reconstruction, we propose four methods to improve the speed and quality of large-scale 3D reconstruction and integrate these algorithms into our system. Figure 1 shows the 3D-reconstruction pipeline of our system.

The data on the input side of the system are images taken by unmanned aerial vehicles (UAVs) from large-scale scenes. In the large-scale-scene sparse point-cloud reconstruction module, the time complexity of image matching is optimized to be approximately linear through the global navigation satellite system (GNSS) neighborhood computing step, which initially filters each image’s neighbors based on spatial location gathered from GPS information. Cluster-based camera graph structure clustering (Section 3.2) and cluster-based global camera pose registration (Section 3.3) are utilized to obtain the global camera pose and perform triangulation and optional bundle adjustment to obtain a sparse point cloud for large-scale scenes.

In the dense point-cloud reconstruction module for large-scale scenes, the two-stage neighborhood image selection method is adopted to ensure that the subsets of neighborhood images are uniformly distributed and that the image content is representative. In addition, GPU parallel depth estimation based on patch matching (Section 3.4) is used to estimate the depth of all views quickly. Finally, the depth map fusion method generates dense point clouds of large-scale scenes.

Our large-scale mesh reconstruction and optimization module obtains the mesh model by using point cloud tetrahedron and surface reconstruction methods, followed by a cluster-based mesh-optimization method for geometric detail recovery (Section 3.5), which produces a mesh model with low redundancy and recovers details from a large-scale scene. We go through several steps in the large-scale-scene texture reconstruction module, including view selection optimization, global color adjustment, global seam processing, and texture atlas generation. As a final step, the system produces a mesh model with texture.

### 3.2. Cluster-Based Camera Graph Structure Clustering

The required number of camera poses grows proportionally with the amount of photos used as input. If all camera poses are directly calculated, a single computing node’s resource limit will be exceeded, and the execution will fail. Additionally, due to the constraints of collecting large-scale-scene data, some cameras have inadequate image-matching relationships, leading to the elimination of these cameras as external points and to a subsequent drop in the quality of the 3D reconstructed models, or to the emergence of holes and other issues. We present a camera graph clustering method based on graph partitioning that clusters all input cameras into multiple camera subsets based on their matching relationship.

Each subset consists of cameras from the same category, ensuring a low correlation across subsets and a high correlation within subsets. Separate camera categories conduct local camera pose estimations in parallel on different cluster computing nodes, ensuring the quality and efficiency of the reconstruction.

#### 3.2.1. Normalized Cut Algorithm

The fundamental rule of camera graph partitioning for large-scale-scene 3D reconstruction is that the number of cameras in each cluster is comparable and controllable. We present a normalized partitioning to handle the problem of clustering camera graph nodes in large-scale scenes. The approach takes into account the partition value and the node’s degree of connectedness, and a normalized cut [80] function is built:

(1) $$Ncut\left(A,B\right)=\frac{cut(A,B)}{assoc(A,V)}+\frac{cut(B,A)}{assoc(B,V)},$$

where $cut\left(A,B\right)=\Sigma_{u\in A,v\in B}w\left(u,v\right)$ is the partition value defined by the minimum cut algorithm and $assoc\left(A,V\right)=\Sigma_{ut\in A,tev}\;w\left(u,t\right)$ is the sum of the connecting edge weights of node A and every other node in the camera network structure. Using the ratio of cut(A,B) to the degree of connection before cut assoc(A,V) as the standard of normalized segmentation can prevent a small number of points from being cut during the minimum partitioning. If a single node is cut to create a subgraph, the ratio equals 1, but it is not the minimum value of the normalized cut value. Consequently, a normalized cut can alleviate the issue that the minimal partitioning is not consistent within subgraphs and is sensitive to external points.

#### 3.2.2. Camera Graph Division

After extracting and matching the image feature points of a large-scale-scene image set, the matching relationship between each image may be determined, allowing the camera graph structure G=(V,E) to be formed, where each vertex in the set *V* represents an image or camera position. The edge connecting two vertices in set *E* demonstrates a matching relationship between two photos. In this study, the number of matching feature points between two pictures is represented by the edge weight $w\left(i,j\right)=|M_{ij}|$, where *i* and *j* represent two nodes.

Using all the nodes, edges and edge weights in the camera graph *G* as input, the normalized cut algorithm may break the camera graph into two subgraphs. Consequently, the original camera graph can be subdivided into *k* subgraphs by conducting normalized segmentation iteratively on the subgraphs. If there are an excessive number of subgraph nodes, the task of computing the camera’s position will surpass the resource limit of a single computing node. Conversely, if there are insufficient subgraph nodes, more image-matching associations will be severed, and more compute nodes would lose data transmission. In light of the use of computational resources and the integrity of the image-matching connection, the segmented subgraph must satisfy the following conditions:

(2) $$\forall G_{i}\in G_{c},\left|V_{i}\right|\leq N_{limit},$$

where $G_{c}$ represents the set of all subgraphs after partitioning, $G_{i}$ and $G_{j}$ stand for the subgraph, $|V_{i}\left|,\right|V_{j}|$ represents the number of vertices in the subgraph, and $N_{limit}$ represents the threshold value of the number of cameras in the subgraph.

Our strategy can fully exploit the benefits of clusters. If the cluster contains *n* compute nodes, the original camera graph should be partitioned into at least *n* subgraphs, and then the normalized cut process should be repeated until the partition requirement is satisfied. The partitioning procedure resembles a tree structure. The normalized cut algorithm guarantees that the number of nodes in each layer is comparable. Each partition selects the subgraph with the greatest number of nodes from the root node.

The original camera graph G=(V,E) can be partitioned into numerous subgraphs *G* based on the cluster resources and the number of input images $G_{i}$ using camera graph partition. All subparagraphs comprise subparagraph set $G_{c}$. Consequently, the existing relationship is $\forall G_{i},G_{j}\in G_{c},\;G_{i}\cap G_{j}=⌀$.

#### 3.2.3. Camera Graph Expansion

The original camera graph is separated into many camera subgraphs once the camera graph is divided and no coincidence is found between them. However, overlapping cameras between subgraphs are required to perform the future 3D-reconstruction task, and the subgraphs after the camera graph partition should be enlarged accordingly. Specifically, each subgraph does not have to share points with all other subgraphs. It is worth noting that the coincidence ratio between subgraphs is guaranteed to allow each subgraph to be fused.

We define the camera block graph structure $G_{seg}=\left(V_{c},E_{c}\right)$, where each node represents a subgraph and the edges between nodes indicate the cut edge set between subgraphs. The weight of an edge is the sum of the weights cut between subgraphs divided by the total number of nodes in the two subgraphs. In a block graph, the weight of the edge connecting subgraphs A and B can be written as:

(3) $$w\left(A,B\right)\;=\frac{cut(A,B)}{|V_{A}\left|+\right|V_{B}|},$$

where cut(A,B) denotes the division between subgraphs *A* and *B*. $|V_{A}|$ and $|V_{B}|$ indicate the number of nodes in subgraphs *A* and *B*, respectively.

In the camera graph expansion, excessive redundancy will develop if each subgraph restores the cut edges between all its surrounding subgraphs. In addition, low-weighted matching relationships are susceptible to wrong camera postures, resulting in faulty camera posture registration. Consequently, based on the concept of a maximum spanning tree, we build the maximum spanning tree of the camera block diagram and extend the camera diagram based on the obtained edges.

First, a weighted undirected graph adjacency matrix that includes all vertices and edges from the original camera graph, i.e., all image-matching associations, is maintained. The elements in the adjacency matrix’s row *i* and column *j* denote the number of image-matching feature points for images *i* and *j*, where there is a value of 0 if no matching relationship occurs. To satisfy the query requirements with constant time complexity, save the adjacency matrix in a hash table.

Secondly, based on the information of the adjacency matrix and the vertex labels in the subgraph, it is simple to obtain all the edges that have been cut as a result of the normalized splitting and apply the inverted approach to rank all the cut edges from a heavy to light weight. By querying the adjacency matrix in pairs using the sequence numbers of all nodes between subgraphs *A* and *B*, it is possible to determine the cut(A,B) values between subgraphs.

Finally, the ratio of cut(A,B) of subgraphs A and B to the total number of nodes in both subgraphs and $|V_{A}\left|+\right|V_{B}|$ is utilized as the edge weight of the camera block diagram, that is, $w\left(A,B\right)=\frac{cut(A,B)}{|v_{A}\left|+\right|v_{B}|}$, ensuring that edges with higher weights have a higher quality image-matching relationship.

Camera graph expansion does not require the restoration of all severed edges. In addition, there may be numerous subgraph extensions whose stop condition is that all the severed edges are restored, resulting in a reduced overall expansion ratio and insufficient matching relationships. Therefore, we define the extension condition as follows:

(4) $$\frac{\Sigma \left|V_{expansion}\right|}{\Sigma |V_{i}|}\;\geq \delta_{ratio},$$

where $\left|V_{expansion}\right|$ represents the number of new vertices that contribute to the expansion, and $|V_{i}|$ represents the number of images present before the expansion of the subgraph. $\delta_{ratio}$ represents the expansion scale, with values typically ranging from 0.5 to 0.7. Expansion stops when the scale of expansion vertices is equal to or greater than that scale, or when all severed edges between subgraphs have been restored.

### 3.3. Cluster-Based Global Camera Pose Registration 

The registration of camera posture is a vital stage in motion recovery structure technology. The precision of the camera pose has a direct effect on the precision of the reconstruction of 3D sparse point clouds. Since the registration of camera poses necessitates simultaneous computation of all camera rotation matrices and translation vectors, it is time-consuming and frequently exceeds the memory limit of computing nodes. This section proposes a quick and precise camera pose averaging approach for combining and optimizing the camera pose of each subgraph.

Similar to [81], a camera pose fusion method based on similarity transformation is employed to unify the local camera poses of the preceding subgraphs in order to generate a global camera pose coexisting in the same world coordinate system. The following function converts between the two camera poses:

(5) $$\left[\begin{array}{cc} R_{j} & T_{j}\\ 0^{T} & 1 \end{array}\right]=\left[\begin{array}{cc} r_{ij} & t_{ij}\\ 0^{T} & 1 \end{array}\right]\left[\begin{array}{cc} R_{i} & T_{i}\\ 0^{T} & 1 \end{array}\right],$$

where $R_{i}$ and $T_{i}$ represent the rotation and translation of camera *i*. $R_{j}$ and $T_{j}$ represent the rotation and translation of camera *j*. The relative rotation and translation between cameras i and j are denoted by $r_{ij}$ and $t_{ij}$, respectively. $R_{j}$ and $T_{j}$ can be inferred to be:

(6) $$R_{j}=r_{ij}R_{i},$$

(7) $$T_{j}=r_{ij}T_{i}+t_{ij}.$$

These calculations demonstrate the conversion between two camera poses. The relative rotation and translation of two cameras can be determined based on their camera poses in their respective world coordinate systems. Using the similarity transformation method, global rotation and global shift fusion are performed, and the combined camera poses are optimized using nonlinear optimization to obtain correct global camera poses.

#### 3.3.1. Global Rotation Registration

After camera graph expansion, the overlapping camera set created by two camera sets $C_{i}$ and $C_{j}$ is denoted as $\left\{C_{rpt}|C_{rpt}=C_{i}\cap C_{j}\right\}$. Initially, using the similarity transformation approach, the relative rotation of each camera in the camera set is computed. The global rotation of the coincidence camera in $C_{rpt}$ is therefore fixed, and the relative rotation of the camera in camera set $c_{j}$, with respect to all other cameras, is utilized. The final formula for calculating the global rotation of all cameras in $C_{j}$ in the coordinate system of $C_{i}$ is as follows:

(8) $$\forall c_{j}\in C_{j},R_{j}^{\prime }=r_{rel}R_{rpt}^{\prime },$$

where $R_{j}^{\prime }$ represents the camera rotation of the cameras in camera sets $C_{j}$ and $C_{i}$ following the fusing of their coordinate systems. $r_{rel}$ represents the rotational relationship between the coincident camera $C_{rpt}$ and the other cameras in the set $C_{j}$. Likewise, $R_{rpt}^{\prime }$ represents the camera rotation of the corresponding camera $C_{rpt}$ in set $C_{j}$.

In our method, the rotation fusion of two camera sets, $C_{i}$ and $C_{j}$, is accomplished by securing a coincident camera, $C_{rpt}$. Consequently, it is necessary to select the most precise coincident camera as the reference camera for the camera rotation fusion of the two camera collections. Since we compute all camera rotations belonging to the camera set, with the exception being the reference camera, some coincident cameras may be calculated several times. These constantly calculated camera rotations serve as the evaluation criteria for inaccuracies. We set $R_{j}^{\prime }$ as the outcome of the calculation for the rotation of coincident cameras, excluding the reference camera.

The rotation of that coinciding camera in camera set $C_{i}$ is set to $R_{i}$, and the error is determined as the Euclidean distance between $R_{j}^{\prime }$ and the $R_{i}$ matrix. Through iterative calculation, a specific reference camera is chosen to minimize the sum of errors between the rotations of all coincident cameras and the initial rotation. Theoretically, we believe the fixed camera to be the most accurate reference camera, and this camera serves as the reference for global rotation and the fusion of the two camera sets.

#### 3.3.2. Global Rotation Registration

Each camera subgraph performs a local estimation of the camera’s position, resulting in translations with varying translation scales. The translation scale has no effect on the global rotational fusion problem described in Section 3.3.1. Nonetheless, the fusion of global translations necessitates the computation of the scale value of the local translation vector in each subgraph. Using the similarity transformation approach, the relationship between the translation vectors of two cameras belonging to a collection of coincident cameras in the same coordinate system can be determined.

(9) $$T_{2}=r_{12}T_{1}+t_{12},$$

(10) $$T_{2}^{\prime }=r_{12}T_{1}^{\prime }+t_{12}^{\prime },$$

where $T_{1}$ and $T_{2}$ are the camera 1 and 2 translation amounts from subgraph 1, and $T_{2}^{\prime }$ and $T_{1}^{\prime }$ are the camera 1 and 2 translation amounts from subgraph 2. $t_{12}$ and $t_{12}^{\prime }$ represent the relative translation between camera 1 and camera 2 in subgraph 1 and 2, respectively. The preceding Equations (9) and (10) describe the translation of two identical cameras in two distinct coordinate systems, and their relative rotation is denoted by $r_{12}$.

All coincident cameras are grouped into $\frac{n(n-1)}{2}$ camera pairs in $C_{rpt}$, where $n=|C_{rpt}|$. Each camera pair’s scale is computed as $\frac{\left|t_{ij}^{\prime }\right|}{\left|t_{ij}\right|}$. Then, based on the following formula, the precise scale of the two cameras’ coordinates is obtained:

(11) $$\lambda_{t}=\frac{\sum_{i,j\in C_{rpt}}\frac{\left|t_{ij}^{\prime }\right|}{\left|t_{ij}\right|}}{|C_{rpt}|}.$$

We directly utilize the reference camera from the global rotation fusion task for the global translation fusion. The reference camera is $T_{a}$ in the current coordinate system and $T_{a}^{\prime }$ in the target coordinate system. $T_{b}$ and $T_{b}^{\prime }$ in the current and target coordinate systems, respectively, represent the camera to be fused. $r_{ab}$ indicates the rotational relationship between the reference camera and the other cameras. The pertinent formula for the camera translation fusion method is as follows:

(12) $$t_{ab}=T_{b}-r_{ab}T_{a},$$

(13) $$T_{b}^{\prime }=r_{ab}T_{a}^{\prime }+\lambda_{t}t_{ab}.$$

First, using Equation (12), we determine the relative shift $t_{ab}$ between the $T_{b}$ of the camera to be processed and the $T_{a}$ of the reference camera. Then, the base camera translation $T_{a}$ is transformed to the target coordinate system translation $T_{a}^{\prime }$. Finally, the global translation vector $T_{b}^{\prime }$ in the target coordinate system is computed using Equation (13) and the obtained translation scale $\lambda_{t}$.

This section discussed the method of fusing two camera subgraphs. Our method does not necessitate overlapping subgraphs but rather ensures that, for each merging, each new subgraph has a coincidence relationship with the merged camera cluster.

#### 3.3.3. Optimization of Camera Poses

If the coarse precision global camera pose information is directly entered into the subsequent reconstruction module following the global camera pose fusion, the reconstruction details will be significantly flawed. Therefore, we optimize all camera poses in the same global coordinate system in order to obtain more precise and reliable global camera poses.

The relative rotation and relative shift obtained by performing local camera pose estimation on each subgraph are saved and recorded as $\left\{R_{rel}\right\}$ and $\left\{T_{rel}\right\}$, respectively. The global rotation and global shift set of all cameras after global camera pose fusion are recorded as $\gamma =\{R_{i}\}$, $\tau =\{T_{i}\}$. The following optimization formulas are defined:

(14) $$arg\;\min_{\gamma }\sum_{r_{ij}\in R_{rel}}d^{R}{\left(r_{ij},R_{j}R_{i}^{T}\right)}^{p},$$

(15) $$arg\;\min_{\tau }\sum_{t_{ij}\in T_{rel}}d^{T}{\left(t_{ij},T_{j}-R_{j}R_{i}^{T}T_{i}\right)}^{p},$$

where $d^{R}$ denotes the angular distance, $d^{T}$ denotes the Euclidean distance, and *P* takes a value of 2 to denote the $l_{2}$ paradigm.

Due to the estimation of the relative rotation and translation based on the local camera location of each subgraph, the relationship between subgraphs can be ensured to have the benefit of normalized segmentation. In other words, the blocks are highly interconnected. Taking these close correlations $\left\{R_{rel}\right\}$ and $\left\{T_{rel}\right\}$ as inputs not only eliminates the need to calculate the relationships between all camera poses but also optimizes the overall global camera pose. The global solid camera pose is derived by optimizing the above formula iteratively. The subsequent reconstruction pipeline generates sparse point clouds by triangulation and obtains more accurate camera pose and sparse point cloud results through bundle adjustment.

### 3.4. GPU Parallel Depth Estimation Based on Patch Matching 

During the dense reconstruction phase of the point cloud, we may still utilize the method outlined previously to compute the smaller dense reconstruction tasks in parallel on each cluster node. The dense point-cloud reconstruction method using depth map fusion is ideally suited for GPU-based parallel computation. Therefore, in this part we propose a GPU parallel fast depth-estimation method for a single computing node, which completely uses the computing resources of a single node and reduces the reconstruction time of dense point clouds on a single computing node.

#### 3.4.1. Random Initialization of the Depth Normal Vector

Our first step is to randomly establish the normal depth vector, whose primary activity is to randomly initialize a collection of possible values for each pixel point in the input reference picture $I_{R}$. Due to the localization of the continuous variation of the image’s photometric information, the problem of estimating the depth value *Z* is transformed into the problem of estimating the support plane *f*, which is described as follows:

(16) $$Z=\;-\frac{d}{n^{T}K_{i}^{-1}p_{i}},$$

where *n* is the normal vector supporting the plane and *d* is the distance between the center of the plane *O* and the center of the reference camera $C_{R}$. It is possible to translate the pixel coordinates $p_{i}={\left[u,v,1\right]}^{T}$ in the ith image to the 3D point $X_{i}$ in the ith camera coordinate system, and its relationship to the depth value *Z* is given by $X_{i}=ZK_{i}^{-1}p_{i}$.

Typically, the depth of a camera-captured image varies nonlinearly. In particular, the depth distribution is dense around the light’s center and sparse the more distant it is from the camera. The depth value conforms to the features of a dense distribution when close and a sparse distribution when far away. The values for parallax and depth satisfy the following formulas:

(17) $$Z=\frac{B\cdot F}{disp}.$$

*B* is the camera baseline distance, and *F* is the focal length. disp is the parallax value, which satisfies the uniform distribution of $U(disp_{min},disp_{max})$. $disp_{min}$ and $disp_{max}$ use sparse point clustering *S* estimates while expanding the value space to increase the range of random initialization values for *d* and to ensure coverage of the entire scene depth range where α is double:

(18) $$disp_{\min}=\max\left(\frac{B\cdot F}{\max_{X\in S}Z}-\alpha \left(\frac{B\cdot F}{\min_{X\in S}Z}-\frac{B\cdot F}{\max_{X\in S}Z}\right),0\right),$$

(19) $$disp_{\max}=\frac{B\cdot F}{\min_{X\in S}Z}+\alpha \left(\frac{B\cdot F}{\min_{X\in S}Z}-\frac{B\cdot F}{\max_{X\in S}Z}\right)).$$

This changes the problem of depth estimation into one of planar parameter estimation. The random initialization method is used as described above to provide a fair distribution of normal vector *n*, which is the distance *d* between the center of the camera and the center of the supporting plane.

#### 3.4.2. Cost Assessment Based on Patch Matching

Next, we analyze possible solutions using an enhanced patch-matching method. A square neighborhood pixel set is defined with the pixel *p* as the center and the radius *r* as the patch *W*. Assuming that all of the pixels in a patch block are on the same support plane *f*, the homography rule is satisfied between the patch blocks of two adjacent images.

Homography is used to map each pixel $p_{R,j}$ in the patch block of the reference image to the matching point $q_{ij}\;=\;H_{i}\left(p_{\mathrm{Rj}}\right)$ in the neighborhood image. This part does not directly calculate the feature difference between two adjacent pixels in order to improve the robustness of the similarity calculation. Instead, to calculate the similarity of patch blocks, the NCC approach is used [50]. Pixels $p_{R,j}$ and $q_{ij}$ should correspond to the same position *X* in the 3D scene if the proposed solution is true. Due to the local nature of the image, the similarity between patches should be strong.

The similarity score computed by NCC is essentially the deformed correlation coefficient. Here, we specify [0, 1] as the value range. To reduce the influence of extreme values, separate weights are provided for each pixel of patch block *W* based on its physical distance from the central pixel and the difference in texture information. This section measures the correlation between pixel *b* and the central pixel *p* in patch block *W* using bilateral weights:

(20) $$w_{b}=\exp\left(-\frac{\left|\left|I(b)-I(p)\right|\right|}{2\sigma_{c}^{2}}-\frac{\left|\left|b-p\right|\right|}{2\sigma_{g}^{2}}\right).$$

As the NCC approach solely evaluates photometric data, it is significantly affected by variations in local illumination. To tackle the problem of similarity measurement failure caused by the change in brightness information resulting from visual angle translation, the picture gradient information item is added to the similarity measurement algorithm.

(21) $$m_{f}\left(p_{i}\right)=m_{f}\left(p_{i},I_{R},I_{i}\right)+m_{f}\left(p_{i},\Delta I_{R},\Delta I_{i}\right).$$

In this section, we estimated the confidence of candidate solutions using the modified NCC technique, which is simple for quick parallel calculations and is stable for measuring the benefits and drawbacks of candidate solutions.

#### 3.4.3. GPU Parallel Depth Map Generation and Optimization

The GPU architecture is an infrastructure that is more suited for multi-core parallel computing, since it has a large number of cores and can handle the simultaneous processing of a huge amount of data as compared to the CPU architecture’s strong scheduling management and coordination capability. Numerous techniques [82,83,84] have adopted the propagation mode of a red-and-black checkerboard grid by partitioning the image pixels into groups via a red-and-black checkerboard, and sampling the adjacent pixels through diffusion.

To limit the influence of extreme values and increase the method’s robustness, the current pixel solution is built using k-candidate solutions with the smallest cost loss. In the adaptive neighborhood, *k* candidate solutions with the lowest cost loss are chosen for each pixel point *p*. Using the cost evaluation method in Section 3.4.2, the cost loss of the *k* candidate solutions in the *n* neighboring view angles is recalculated to yield the loss matrix *M*.

It can be assumed that a particular view angle of pixel *p* does not satisfy the photometric consistency assumption due the presence of object occlusion and similar conditions. In such a scenario, the cost losses of *k* potential options become considerable. In contrast, there will be at least one candidate solution with a minor cost loss from an excellent neighborhood perspective, and the cost loss from a good neighborhood perspective will decrease gradually as the number of iterations increases. The permissible cost’s upper bound for iteration *t* is defined as follows:

(22) $$TG\left(t\right)=TG_{0}\cdot e^{-\frac{t^{2}}{\psi }},$$

where $TG_{0}$ is the upper bound of the initial cost and ψ is a constant. The threshold TB is defined as the greatest cost loss and the cost loss $m_{ij}$ is the *i*-th potential solution from the *j*-th perspective. If $m_{ij}<TG\left(t\right)$ is matched, the *i*-th candidate solution is a superior solution from the *j*-th perspective, and it is added to the set SG(j). If $m_{ij}>TB$ is matched, the *i*-th candidate solution is added to the set SB(j) as an incorrect solution at the *j*-th view. If the number of better solutions of the *j*-th view is greater than $n_{1}$ and fewer than $n_{2}$, we consider the *j*-th view to be the better neighborhood image of the pixel in the *t*-th iteration and add it to the neighborhood image set $S_{t}$ of the pixel.

Despite the fact that photos with uneven brightness are eliminated by setting the threshold TB, each neighborhood image will contribute varied weights to the results due to noise, scale and other variables. The following weights are applied to each neighborhood image, given that β is a constant.

(23) $$w\left(I_{j}\right)=\frac{1}{\left|SG(j)\right|}\sum_{m_{ij}\in SG\left(j\right)}e^{-\frac{m_{ij}^{2}}{2\beta^{2}}},I_{j}\in S_{t}.$$

Due to issues, such as occlusion, weak texture and lighting conditions, it is impossible to ensure the correctness of the depth map estimate in natural scenes when solely using the photometric consistency assumption. In light of this, we introduce the geometric consistency assumption while maintaining the photometric consistency assumption. It is believed that the 3D point recovered using the depth information of the pixel *p* in the reference image is identical to the 3D point restored using the depth information of the corresponding point $p^{\prime }$ in the neighboring image.

We employ the reprojection error metric to satisfy the degree of geometric consistency constraint, assuming the depth of the pixel *p* equals $D_{R}\left(p\right)$. The formula for the point $q_{j}$ corresponding to the j-th neighborhood image is as follows:

(24) $$q_{j}=P_{j}R_{R}^{T}\left(D_{R}\left(p\right)\cdot p\;-\;t_{R}\right).$$

To obtain a pixel $p_{j}^{\prime }$, the pixel $q_{j}$ projection that refers the image back in the same manner based on its depth value $D_{i}\left(q_{j}\right)$ is:

(25) $$p_{j}^{\prime }=P_{R}R_{j}^{T}\left(D_{i}\left(q_{j}\right)\cdot q_{j}\;-t_{j}\right).$$

The definition of the reprojection error $e_{ij}$ of the *i*-th candidate solution at the *j*-th viewing angle is:

(26) $$e_{ij}=\min\left(∥p_{j}^{\prime }-p∥,\delta \right).$$

In order to improve the robustness of the reprojection error and tackle the excessive error generated by masking and other factors, it is necessary to increase the error resistance. We select the ideal solution from the candidate solutions based on the photometric geometric consistency cost $c_{i}$, where μ is a constant.

(27) $$c_{i}=\frac{\sum_{j=1}^{N}\tilde{w}\left(I_{j}\right)\cdot \left(m_{ij}\;+\mu \cdot e_{ij}\right)}{\sum_{j=1}^{N}\tilde{w}\left(I_{j}\right)}.$$

From the *k* candidate solutions, the average value of the *n* candidate solutions with the lowest cost of photometric geometric consistency is chosen as the new solution. During the entire photometric geometric consistency-solving procedure, only the current pixel and the candidate solution pixel are utilized. The parallel calculation at the pixel level can be performed by combining the red-and-black checkerboard sampling method described in Section 3.4.1.

Using the random initialization method described in Section 3.4.1, we reintroduce a set of random solutions $s^{rnd}=\left(n^{rnd},d^{rnd}\right)$ for each pixel. The random solution is independent of any prior knowledge. It is chosen with equal probability over the whole solution space, essentially preventing the approach from settling on the optimal local answer. Using the perturbation method, we construct another set of perturbed solutions $s^{prb}=\left(n^{prb},d^{prb}\right)$. The disturbance solution is dependent on the now-achieved ideal solution, and the result is refined by searching in close proximity to the optimal solution.

Then, we utilize the Eulerian angle to represent the rotation disturbance of the normal vector and uniformly sample the Eulerian angle within [−0.5φ,0.5φ] to produce the disturbed normal vector $n^{prb}=R^{prb}\cdot n$. Finally, using the random solution, the perturbed solution and the current solution, we recombine them into nine new solutions as follows:

(28) $$\left(n,d\right),\left(n,d^{prb}\right),\left(n,d^{rnd}\right),\left(n^{prb},d\right),\left(n^{prb},d^{prb}\right),\left(n^{prb},d^{rnd}\right),\left(n^{rnd},d\right),\left(n^{rnd},d^{prb}\right),\left(n^{rnd},d^{rnd}\right).$$

The preceding nine solutions are candidate solutions chosen in Section 3.4.2, and the *k* value is set to nine. The multi-candidate solution joint depth-estimation method is re-executed, and the final solution provided is considered the method’s optimal solution.

### 3.5. Cluster-Based Mesh Optimization for Geometric Detail Recovery

We rebuilt large-scale 3D mesh models using a mesh generation method [85] based on Delaunay point cloud tetrahedralization. Due to redundant data, an uneven structure, a high level of spatial noise and imprecise mesh details, this section provides a mesh-optimization strategy for large-scale mesh scenarios based on cluster geometry detail recovery. The method consists of three main steps: the first step is mesh simplification, which focuses on maintaining the model’s detailed features while minimizing the mesh model’s redundancy.

The second step is mesh smoothing. To effectively eliminate the noise and outliers of the mesh, we propose a mesh-smoothing homogenization method based on the second-order umbrella operator, which makes the mesh regular and homogeneous. The last step is mesh-detail recovery. As a result of combining the image color-consistency term and the Laplace smoothing term, we construct an energy function that further removes noise and outliers while recovering more detailed features of the mesh.

#### 3.5.1. Mesh Simplification

To ensure that the generated mesh model meets the accuracy requirements, the reconstruction process as a whole uses a very high resolution to retain as much of the input information as possible. This causes the data amount of the generated mesh model to increase rapidly as the reconstruction range expands. Nonetheless, a significant number of redundant meshes will cause the subsequent texture reconstruction to take an excessive amount of time and memory. On the basis of maintaining the accuracy and realism of the grid model, the complex mesh model will be replaced with a reduced mesh model, thereby enhancing the model’s operational efficiency.

The majority of mesh simplification algorithms [59,60,61,62] not only lower the amount of model data, but also fail to preserve the model’s detailed features, resulting in a reduction in the mesh model’s realism. Consequently, the complexity of different sections of the mesh model must be taken into account, and varying degrees of simplification are applied based on the region’s characteristics in order to preserve the model’s details.

Inspired by Jiang et al. [65], we employ a feature-preserving-based mesh-simplification method, which differs from the QEM mesh-simplification method in that the semantic information of the mesh vertices, the vertex first-order neighborhood triangular patch area, the approximate vertex curvature, the average shape of the vertex first-order neighborhood triangular patch, and the position of the vertex pair in the model are introduced to enhance the new vertex replacement cost calculation method. Simultaneously, the replacement cost of new vertex pairs located on the local detail-rich surface of the model and the vertex pairs located on unrelated objects is increased, thereby preventing these vertex pairs from being selected as simplified objects and preserving the local characteristics and authenticity of the mesh model.

#### 3.5.2. Mesh Smoothing

Although the mesh-smoothing method based on Laplace’s theorem can reduce the roughness of the model surface, make the mesh model surface smooth and effectively reduce the impact of noise points on the model quality of dense point-cloud data, there is a more effective way to reduce the impact of noise points on the model quality. However, after multiple iterations of this procedure, the model will become more visibly deformed, and the mesh model will decrease, diminishing the realism of the object.

We present an approach for smoothing homogenization based on a second-order umbrella operator. The fundamental idea is to modify the coordinates of the vertices by referencing the position adjustment of vertices in the first-order neighborhood to minimize the model distortion that results from the adjustment of vertex coordinates. The movement direction of the vertex is closer to the projected gradient along the vertex’s corresponding tangent plane, resulting in a more regular and uniform triangular patch.

In the mesh-smoothing approach, the Laplacian smoothing algorithm has a high operation efficiency, and the smoothing process applies the umbrella operator to the mesh iterative vertex movement. The computation procedure can be expressed by the following equation:

(29) $$\frac{\partial S}{\partial t}=\nabla^{2}S,$$

where ∂S is the input initial mesh model to be processed and $\nabla^{2}$ is the iteratively moving vertex constraint term. The iterative process, i.e., the Laplace operator, can be stated using the following equation:

(30) $$S_{t+1}=S_{t}+\lambda \nabla^{2}S_{t},$$

where *t* indicates the number of smoothing rounds, $S_{t}$ represents the mesh model after *t* rounds of smoothing, and λ represents the radical smoothing degree. A large value suggests a more extreme mesh-smoothing degree. As demonstrated in the following equation, the 3D Cartesian coordinates of each mesh vertex are converted into the appropriate Laplace coordinates U(v).

(31) $$U\left(v\right)=\frac{1}{\sum w_{k}}\sum^{}w_{k}v_{k}-v\;\;k\epsilon NV_{1}\left(v\right),$$

where *v* represents the 3D Cartesian coordinates of the mesh model’s vertices and U(v) indicates their corresponding Laplace coordinates. $V_{k}$ indicates a vertex in the vertex’s first-order neighborhood. $NV_{1}\left(v\right)$ is the set of all vertices in vertex *v*’s first-order neighborhood. $W_{k}$ represents the weight of vertex $V_{k}$ in the calculation of the Laplace coordinates of vertex *v*. $\frac{1}{\Sigma w_{k}}$ is used to normalize the weighted combination of the vertices’ 3D coordinates in the first-order neighborhood.

Since several iterations of the Laplacian mesh-smoothing approach are employed, the model will undergo evident deformation, and the mesh model will contract as a whole. We describe a method for mesh smoothing and homogenization based on a second-order umbrella operator that can solve mesh deformation and contraction issues and make the mesh transition between different regions smoother. Our proposed second-order umbrella operator is depicted in Equation (32).

(32) $$U^{2}\left(v\right)=\frac{1}{\sum w_{k}}\sum w_{k}U\left(v_{k}\right)-U\left(v\right)\;\;\;\;k\epsilon NV_{1}\left(v\right),$$

where $U^{2}\left(v\right)$ is the second-order umbrella operator and the weight $W_{k}$ of the vertex $V_{k}$ in the first-order neighborhood is set with the cotangent weight and normalized by $\frac{1}{\Sigma w_{k}}$. Equation (33) illustrates the improved iterative computation approach for vertex coordinates.

(33) $$v_{t+1}=v_{t}+\lambda \left(\rho U\left(v_{t}\right)+\left(1-\rho \right)U^{2}\left(v_{t}\right)\right).$$

When the parameter ρ is less than 1, it is considered a balance parameter. A greater value of ρ implies that each iterative calculation tends to lessen the interference of noise points, resulting in a smoother surface on the mesh model. A decreasing value suggests that each repeated calculation tends to produce a mesh model of triangular patches that is more uniform and regular.

#### 3.5.3. Mesh-Detail Recovery

The mesh-simplification approach and the mesh-smoothing method can improve the mesh quality of the model by eliminating mesh redundancy; however, they only process the details of the mesh’s features, which may result in a mesh that lacks visual realism. In this phase, we optimize the mesh vertex position adjustment and restore the model with more details, which is one of the most crucial steps and improves the quality of the final created model.

To eliminate the noise of the initial mesh and restore the mesh’s detail features, we utilize the photo color consistency and Laplacian smoothing approach to develop an energy formula that comprises the following data items and smoothing terms:

(34) $$E\left(S\right)=\sum_{k=1}^{k}E_{photo}^{k}+\lambda E_{smoothness},$$

where $E_{photo}^{k}$ is the k-th image dataset’s data item and λ(λ≥0) is the smoothing term’s weight. To minimize the energy equation, we generate a gradient vector corresponding to the mesh’s vertices and shift the vertices to the gradient vector until convergence. The image data item is constructed according to the image’s color consistency and is meant to restore the mesh’s intricate details. The smoothing term is derived from the Laplacian operator in order to reduce the image’s noise and improve the quality of the mesh.

The picture data item is produced based on the image’s color consistency. Our goal is to minimize the image reprojection error between image pairings by projecting the pixel points from image $I_{i}$ onto image $I_{j}$ and then calculating the total color consistency of the pixel point and its nearby pixel points. Consequently, this data item is formatted as follows:

(35) $$E_{photo}\left(S\right)=\sum_{k=1}^{K}\sum_{i,j}\int_{D_{ij}^{s}}h\left(I_{i},I_{ij}^{S}\right)\left(x_{i}\right)dx_{i}\;i,j\in Dlock\left[k\right],$$

where $I_{ij}^{S}=I_{j}\circ P_{j}\circ P_{i}^{-1}$ is the reprojection of image $I_{i}$ onto image $I_{j}$ through the mesh and $P_{j}$ and $P_{i}^{-1}$ are the processes of projecting and back-projecting image I onto the mesh, respectively. $h(I_{i},I_{ij}^{S})$ is a decreasing function of the color consistency at pixel $x_{i}$ of images $I_{i}$ and $I_{j}$. $D_{ij}^{S}$ is the region where the image $I_{j}$ is reprojected to the image $I_{i}$. Equation (35) describes the degree of correspondence between the two images and the triangular mesh. The greater the mesh inaccuracy, the greater the $E_{photo}\left(S\right)$ value.

Equation (35) must be discretized because it is continuous while the mesh is discrete. Assuming the two pictures are $I,J:D\to R_{n}$ and $H\left(I,J\right)=\int_{D}h\left(I,J\right)\left(x\right)dx$ and $\Phi_{ij}\left(s\right)=H\left(I_{i},I_{ij}^{s}\right)$, the following equation may be derived:

(36) $$E_{photo}\left(S\right)=\sum_{k=1}^{K}\sum_{i,j}\Phi_{ij}\;i,j\in block\left[k\right],$$

(37) $$\nabla E_{photo}\left(S\right)\equiv \sum_{k=1}^{K}\sum_{i,j}\nabla \Phi_{\mathrm{ij}}\;i,j\in Dlock\left[k\right].$$

Any vertex x∈S can be viewed simultaneously by the images *I* and *J*. Images *I* and *J* are matched pairs that satisfy the equation below:

(38) $$\frac{dP_{i}\left(x\right)}{dx}=-\frac{N^{T}c_{i}dx}{z_{i}^{3}},$$

where *N* is the normal vector of the point *x* perpendicular to the outward direction of the curved surface, $c_{i}$ is the vector of the camera’s center facing point *x*, and $z_{i}$ is the depth of x in the camera *i*’s coordinate system. According to Equation (38), the following formula can be derived:

(39) $$\nabla \Phi_{ij}\left(x\right)=-\frac{\partial H\left(P_{i}\left(x\right)\right)JaI_{j}\left(P_{j}\left(x\right)\right)JaP_{j}\left(x\right)c_{i}N}{z_{i}^{3}},$$

where *H* is the abbreviation for the $H(I_{i},I_{ij}^{S})$ function, Ja is the function of the Jacoby matrix, and H(I,J) is the partial derivative of function ∂H(I,J) with respect to image *J*. According to the aforementioned Formula (39), for every vertex $v_{i}\in S$, the barycenter coordinate of the triangle is $x=\sum \;v_{i}\phi_{i}\left(x\right)$.

(40) $$\frac{dE_{photo}\left(S\right)}{dv_{i}}=\int_{S}\phi \left(x\right)\sum_{k=1}^{K}\sum_{i,j}\nabla \Phi_{ij}\left(x\right)dx\;,i,j\in block\left[k\right].$$

Equation (40) computes the gradient of each vertex corresponding to the discrete triangular mesh in order to change the vertex location along the gradient direction. The appeal process reduces the inaccuracy of the picture color consistency, increases the degree of matching between the image and the mesh and then restores the detail features of the mesh.

Since errors remain in the obtained picture data, we add a smoothing component based on the image color consistency to reduce the influence of image noise on the mesh and boost the mesh’s smoothness. The calculation is as follows:

(41) $$E_{smoothness}\left(S\right)=\int_{S}\left(k_{1}^{2}+k_{2}^{2}\right)dS,$$

where $k_{1}$ and $k_{2}$ are the major curvatures of the mesh surface at the same vertex and the smoothing term measures the entire mesh’s curvature to make it smooth. We construct the smoothing term using the Laplacian smoothing approach. Since the mesh was already smoothed as described in Section 3.5.2, we merely employ the more efficient first-order umbrella operator. The final gradient vector and iterative formulas are as follows:

(42) $$\nabla v_{i}=\frac{dE_{photo}\left(S\right)}{dv_{i}}+\lambda U\left(v_{i}\right),$$

(43) $$v_{i}^{t+1}=v_{i}^{t}+\xi \nabla v_{i}.$$

In Equation (43), ξ represents the increment size. We can move the vertices iteratively in accordance with the equation until the requirement $\frac{\Sigma_{i}^{m}\left|\nabla v_{i}\right|}{m}<\eta$ is satisfied. The iteration is convergent and restores every detail feature in its entirety.

## 4. System Evaluation and Analysis

### 4.1. System Configuration

This research proposes a cluster-based method for the reconstruction of large-scale 3D scenes. In the process of software verification, we assembled a cluster of nine servers. As the cluster’s master node, the server with the highest performance is responsible for the assignment of reconstruction tasks, the distribution of input data and the recovery of intermediate or final output data. As slave nodes, the remaining eight servers are responsible for performing the computing duties assigned by the master node.

To demonstrate the universality of the hardware, we selected two distinct server kinds as child nodes. Table 1 depicts the hardware environment of each cluster node in our experiment. Theoretically, our system has a high degree of flexibility, as it can use a single server or a cluster of multiple servers (where the number of servers is greater than or equal to two) to run the reconstruction system. Table 2 describes the unified program running environment configured on each computing node throughout the cluster.

**Figure 2:**
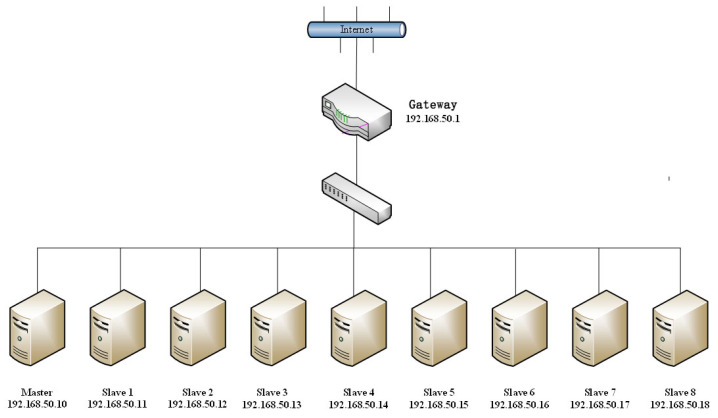
Cluster network topology.

According to the network configuration depicted in Figure 2, we utilized one router, one switch and nine servers. During this time, the SSH key of the mast node was stored in a list of authorized trustworthy servers of each slave node, and communication and data transmissions were achieved using an SSH secret-free login between each server.

### 4.2. System Reconstruction Results

**Figure 3:**
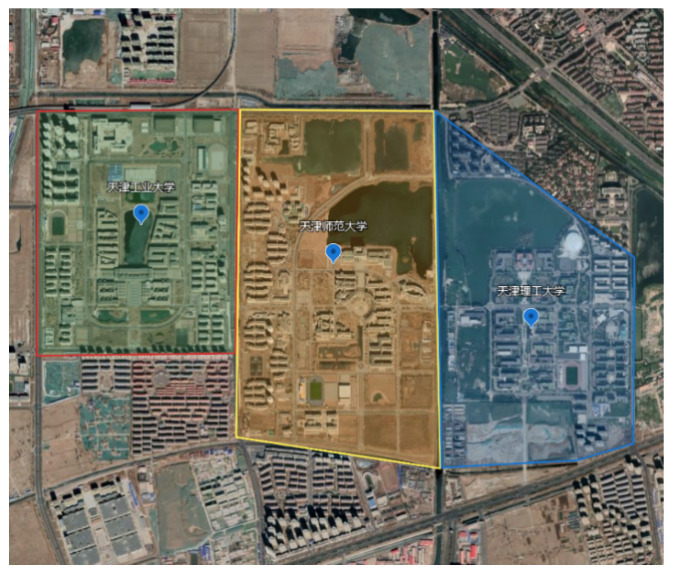
The TJUT dataset [86] aerial photography area marked on Google Maps.

The experimental dataset used was the TJUT dataset [86], which was collected from the university city of Xiqing District, Tianjin (as shown in Figure 3), which contains three campuses with continuous scenes and multiple categories: Tianjin Polytechnic University, Tianjin Normal University and Tianjin University of Technology. A number of DJI Inspire 2 commercial UAVs were used to conduct cooperative photography for this dataset. All UAVs used the same flight and camera parameters. The TJUT dataset contains 38,775 aerial images, each with a resolution of 4000 × 3000, which covers an area of 6.2 square kilometers and includes buildings, roads, playgrounds, vegetation, rivers and other objects.

**Figure 4:**
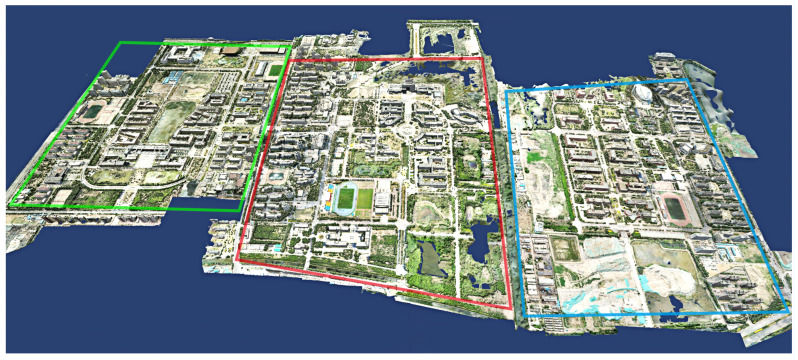
Our system reconstructs a 3D model of the university city of Xiqing District in Tianjin. The green box on the left is Tianjin Polytechnic University with an area of about 1.8 square kilometers. The middle red box represents Tianjin Normal University with an area of about 2.3 square kilometers. The blue box to the right is Tianjin University of Technology with an area of about 2.1 square kilometers.

**Figure 5:**
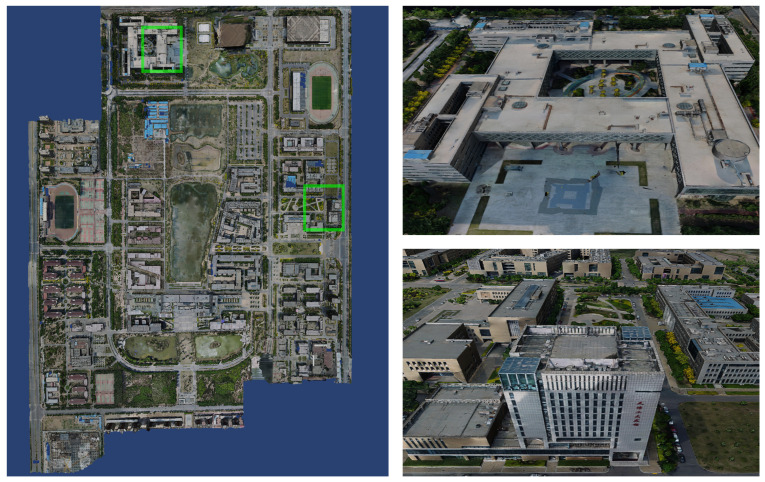
The left side is the global forward view of the 3D model of Tianjin Polytechnic University. The right side shows the details of the model at the location of the green box in the 3D model of Tianjin Polytechnic University.

**Figure 6:**
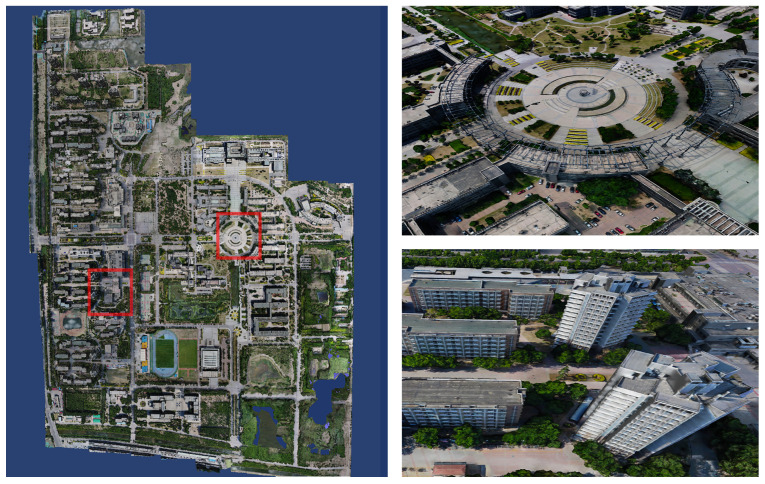
The left side is the global forward view of the 3D model of Tianjin Normal University. The right side shows the details of the model in the close-up view from the red box’s position of the 3D model of Tianjin Normal University.

**Figure 7:**
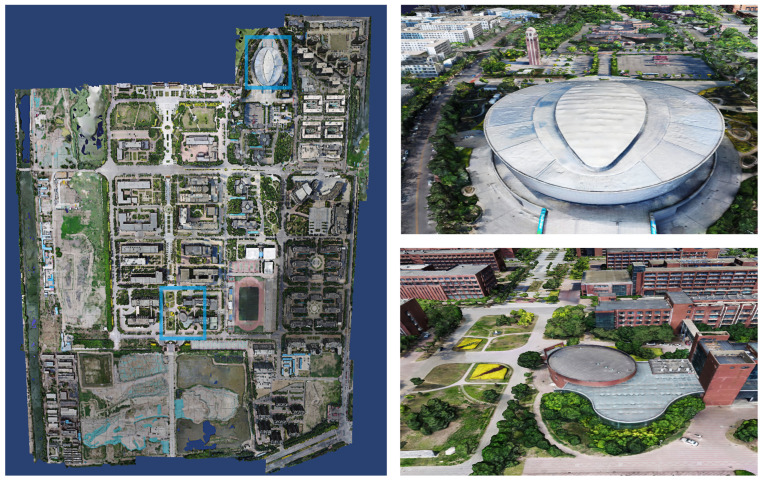
The left side is the global forward view of the 3D model of Tianjin University of Technology. The right side shows the details of the model in the close-up view from the blue box’s position of the 3D model of Tianjin University of Technology.

We set the input path of the dataset and the output path of the model in sequence using the system one-key mode. Set the rebuild quality to standard mode, the number of clusters to 9 and the task weight of all calculation nodes to 1. As soon as the system settings are completed, click Start Reconstruction to reconstruct the textured mesh model of the large-scale scene in its entirety without any artificial intervention. As shown in Figure 4, the system reconstructs the 3D model of the corresponding university town. Figure 5, Figure 6 and Figure 7 demonstrate the 3D models of the three campuses of Tianjin Polytechnic University, Tianjin Normal University and Tianjin University of Technology with a complete vertical perspective alongside details of the 3D models from a close-up perspective.

**Figure 8:**
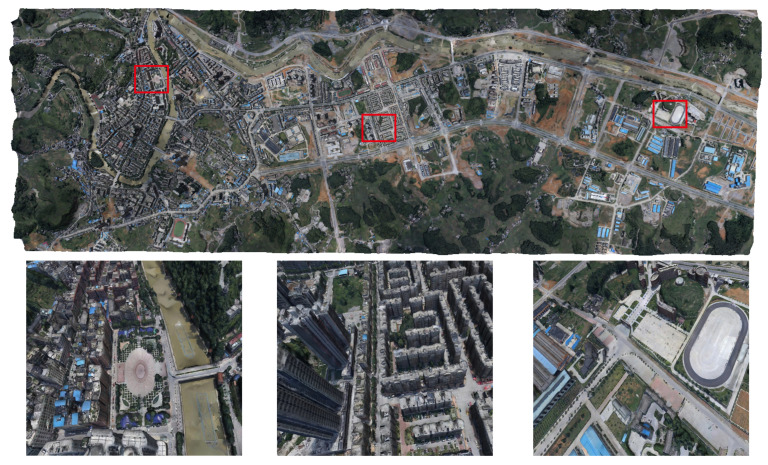
The upper side shows the complete global model reconstructed from the entire WUH dataset [87]. The lower side shows the model details of the close-up view selected from the global scene.

We tested our system on the publicly available WUH dataset [87], which consists of 1776 aerial images with a resolution of about 5376 × 5376 pixels and covers about 14 square kilometers. We used the system’s one-click mode to reconstruct a complete model of the whole scene. As shown in Figure 8, the top half of the figure displays the complete scene model. The bottom half shows the details of the 3D model in three close-up views.

### 4.3. System Comparison

One essential requirement for large-scale 3D scene reconstruction is that the reconstruction speed is as fast as possible to ensure a certain quality so that the reconstructed model can be quickly deployed in many application scenarios, such as disaster rescue and geological surveying. Thus, we compared our system with the mainstream open-source libraries OpenMVG [76] combined with OpenMVS [77], Colmap [78] combined with OpenMVS, and with the mainstream commercial software Pix4Dmapper and ContextCapture. This comparison was made with the same dataset and the same hardware environment.

We split the TJUT dataset into three sub-datasets, TPU (11,245 photos), TNU (14,347 photos) and TJUT (13,183 photos), by region to reduce the amount of input data and make comparisons easier. Since the open-source library OpenMVG + OpenMVS and the commercial software Pix4Dmapper cannot use clusters, the open-source library Colmap + OpenMVS and the commercial software ContextCapture were used because they can use clusters.

To ensure as much fairness as possible, we used the hardware configuration presented in Table 1 with the open-source library OpenMVG + OpenMVS and the commercial software Pix4Dmapper running on the master computing node alone. The open-source library Colmap+OpenMVS and the commercial software ContextCapture used a cluster consisting of three units: master, slave1 and slave6 for reconstruction operations. All open-source libraries and commercial reconstruction software programs were used with the default or medium-quality reconstruction settings. Additionally, we used our system’s fast and standard modes to reconstruct each dataset separately.

The details are shown in Table 3. All libraries and software systems that support clustering outperformed the libraries and software systems that could only execute on a single machine in terms of reconstruction speed. Theoretically, if more compute nodes are added to the cluster, the reconstruction speed will be improved even more. Our system outperformed the same type of open-source libraries and software systems in regard to the reconstruction speed in both standard and fast modes. Note that the fast mode significantly improves the reconstruction speed at the expense of reconstruction quality. In practical applications, it meets the needs of users with urgent time requirements, helping them to quickly obtain preliminary models and grasp the actual data and situation of large-scale scenarios as early as possible.

We conducted a 3D-reconstruction speed-performance evaluation on the WUH dataset. In the same hardware-configuration environment as before, only open-source libraries and commercial software supporting standalone machines used master computing nodes. The open-source libraries and commercial software that support a cluster environment used a cluster of master, slave1 and slave6 for 3D-reconstruction operations. Table 4 shows that our system outperformed the same type of open-source library and commercial software systems regarding the reconstruction speed in both standard and fast modes.

## 5. System Usage Information

This section demonstrates the use cases of the simple and expert modes in our reconstruction system from the user’s perspective. Simple mode means that the user does not need any expertise and only needs to set the input and output paths, work paths, cluster task-assignment parameters and quality parameters. This enables the user to complete partial and overall reconstruction tasks. Expert mode is meant for users that have some expertise in reconstruction algorithms and can set detailed parameters for each reconstruction job to complete a professional custom reconstruction project.

### 5.1. Simple Mode

Our system can complete sparse point-cloud reconstruction, dense point-cloud reconstruction, mesh reconstruction, mesh optimization, texture reconstruction and the entire 3D-reconstruction process. It can also complete the subprocesses of the reconstruction process individually. In simple mode, let us take sparse point-cloud reconstruction as an example. First, open the “Sparse Reconstruction—Automatic Sparse Reconstruction Parameter Settings” dialog box and enter the input, output and project work file paths in order as required.

**Figure 9:**
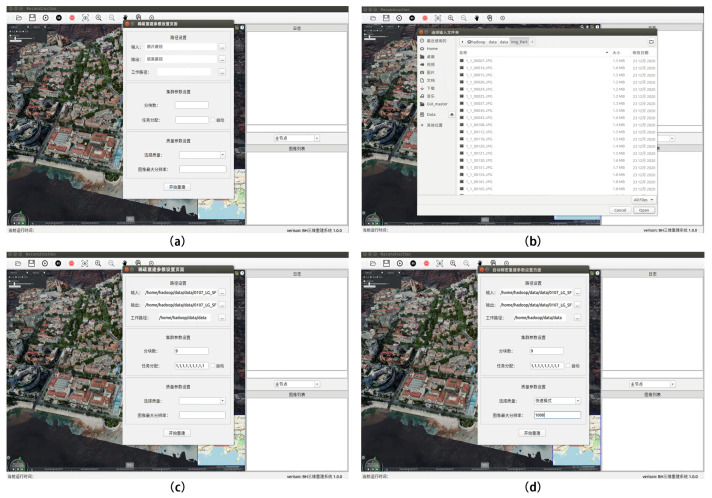
A demonstration of the fully automated 3D sparse point-cloud reconstruction using simple mode (one-click reconstruction). (**a**) sparse reconstruction parameter setting, (**b**) select input folder, (**c**) cluster parameter setting and (**d**) quality parameter setting.

Next, set the number of cluster chunks and the task allocation ratio of each computing node in the cluster. Finally, set the reconstruction quality and the resolution parameters of the image during the reconstruction process. Then, click Rebuild to fully and automatically reconstruct the point-cloud model without any human intervention. The specific operations are shown in Table 5, and Figure 9 shows the demonstration results of the associated operations.

### 5.2. Expert Mode

The system can provide a one-click 3D-reconstruction mode for beginner users in simple mode, whereas expert mode is for professional users who have mastered 3D-reconstruction algorithms. Expert mode provides detailed parameter settings for sparse point-cloud reconstruction, dense point-cloud reconstruction, mesh reconstruction, mesh optimization, and texture reconstruction subprojects throughout the 3D-reconstruction process. Professional users can manually adjust the parameters of each reconstruction subproject to meet their needs, including scene timeliness and scene 3D-reconstruction fineness.

Let us take sparse point-cloud reconstruction as an example. First, click “Sparse Reconstruction—Feature Point Extraction” in the menu bar to automatically bring up the “Feature Point Extraction” dialog box and enter the relevant parameters to control the feature-point extraction quality in the sparse point-cloud reconstruction process. Secondly, select “Sparse Reconstruction—Feature Point Matching” in the menu bar, and fill in the relevant parameters to effectively control the matching quality between image pairs. Again, select “Sparse Reconstruction—BA” in the menu bar and fill in the appropriate BA parameters in the “BA” dialog box to adjust the nonlinear model optimization.

**Figure 10:**
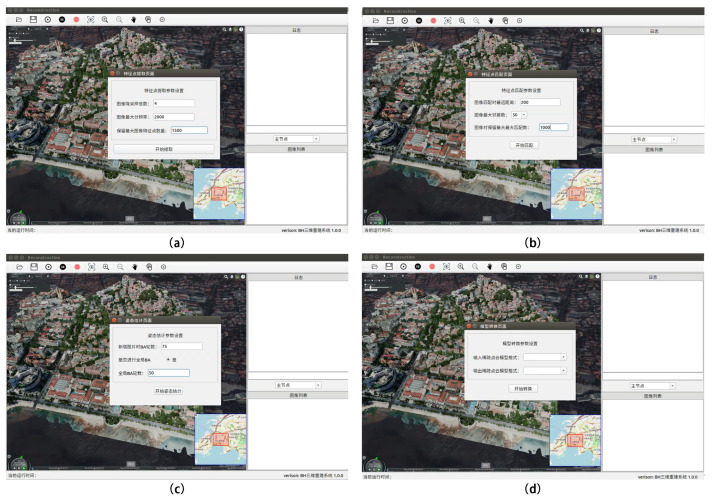
In expert mode (professional parameter setting), the sparse point-cloud 3D-reconstruction example is completed by setting parameters in four steps: (**a**) feature-point extraction, (**b**) feature-point matching, (**c**) bundle adjustment and (**d**) model format conversion.

The final step is to choose “Sparse Reconstruction—Model Conversion” from the menu bar. In the model conversion dialog box, the user can choose the output sparse point-cloud model format for reconstruction (the input is not chosen). Alternatively, the user can convert the sparse point-cloud format by selecting the input sparse point-cloud format and converting it to the output sparse point-cloud format. The detailed operating instructions for expert mode are presented in Table 6, and Figure 10 illustrates the most significant operating steps.

## 6. Conclusions

We developed a cluster-based system for large-scale 3D reconstruction. We proposed cluster-based camera-graph-structure clustering and cluster-based global camera pose-registration methods with a partitioning strategy, which helps to avoid the limitations of a single computing node and can handle 3D-reconstruction tasks of large-scale scenes within the framework of clusters.

In order to fully utilize the computational resources on a single computational node, we developed a GPU parallel depth estimation based on a patch-matching method that uses GPU computational units to achieve the pixel-level parallel computation of depth values, which significantly reduces the time needed to solve the dense point-cloud reconstruction problem on a single computational node. We propose a 3D mesh-optimization method based on cluster geometric detail recovery, which not only effectively reduces the size of the data but also maintains the local characteristics and realism of the model.

Our system can efficiently and rapidly generate large-scale-scene 3D models in both standard and fast modes and outperformed state-of-the-art 3D-reconstruction libraries and commercial systems in terms of the reconstruction speed. In the future, we plan to continue to enhance our system by adding functions to generate digital orthophoto maps (DOM), digital surface models (DSMs) and track-planning and model-accuracy reports. We will continue to improve the reconstruction algorithm of the system to achieve higher reconstruction accuracy and faster reconstruction speed.

## Figures and Tables

**Table 1:** Cluster environment for computing the unit hardware configuration.

Calculation Node Name	CPU	Graphics Card	Memory
Master	Intel(R) Xeon(R) Gold 3160 CPU @2.10 GHz	Titan RTX	256 GB
Slave1~Slave5	Intel(R) Core(TM) i7-8700K CPU@3.70 GHz	GeForce GTX1080Ti	32 GB
Slave6~Slave8	Intel(R) Xeon(R) Sliver 4110 CPU @2.10 GHz	Quadro P6000	64 GB

**Table 2:** Software configuration of a cluster-based large-scale 3D reconstruction system.

System Dependency Library	Version Information
Ubuntu	16.04
Eigen	3.2.10
Ceres solver	1.10
C++	14
OpenCV	3.4.11
CGAL	5.0.1
VCGLib	1.0.1
Boost	1.76

**Table 3:** Comparison of our system with mainstream open-source libraries and commercial software in terms of the 3D-reconstruction speed for the same scene dataset.

			3D Reconstruction Time Consumed (h)		
**Library/Software**	**Version**	**Cluster**	**Dataset 1: TPU**	**Dataset 2: TNU**	**Dataset 3: TJUT**
OpenMVG [76] + OpenMVS [77]	V2.0, V2.0	✘	93.33	123.50	110.75
Colmap [78] + OpenMVS	V3.7, V2.0	✔	68.25	82.58	78.83
Pix4Dmapper	V4.3.9	✘	90.50	110.50	101.67
ContextCapture	V4.6.10	✔	52.17	64.33	59.75
Ours (standard mode)	V1.0.2	✔	48.50	55.75	52.83
Ours (fast mode)	V1.0.2	✔	23.45	27.42	25.92

**Table 4:** The 3D-reconstruction speed performance of our system as compared with other open-source libraries and commercial software, which was conducted using the publicly available WUH dataset [87].

			3D Reconstruction Time Consumed (h)
**Library/Software**	**Version**	**Cluster**	**Dataset: WHU**
OpenMVG + OpenMVS	V2.0, V2.0	✘	69.5
Colmap + OpenMVS	V3.7, V2.0	✔	34.67
Pix4Dmapper	V4.3.9	✘	64.25
ContextCapture	V4.6.10	✔	26.75
Ours (standard mode)	V1.0.2	✔	24.33
Ours (fast mode)	V1.0.2	✔	10.83

**Table 5:** Example of fully automated 3D sparse point-cloud reconstruction by a user using simple mode.

Operation No.	Operating Steps
1	On the system home page, click on “Sparse Reconstruction—Sparse Reconstruction Parameter Setting”, as shown in Figure 9a.
2	As shown in Figure 9b, click the folder selection button on the right side of “Enter image path” to select the path of the input image folder.
3	Select a storage folder path for the output sparse point-cloud model by clicking the folder selection button on the right side of “Output Path”.
4	To select the storage path for the intermediate files, click the folder selection button on the right side of the “Work Path”.
5	Set the number of chunks in the reconstruction area—that is, set the number of compute nodes in the requested cluster.
6	Figure 9c shows how to set the task allocation ratio of each calculation node or select the check box to allocate tasks evenly by default.
7	Click the quality selection combo box and select the desired quality of the 3D reconstruction.
8	Set the image resolution used in the sparse point-cloud reconstruction.
9	As shown in Figure 9d, click the Start Reconstruction button to begin the fully automated, hands-free process of 3D sparse point-cloud reconstruction.

**Table 6:** Example of setting sparse point cloud reconstruction parameters in expert mode.

Operation No.	Operating Steps
1	On the system home page, the user selects “Sparse Reconstruction-Feature Extraction” in the menu bar, and the “Feature Extraction” dialog box appears.
2	As shown in Figure 10a, set the downsampling multiplier, the maximum resolution, and the maximum number of retained image features based on the requirements of the user.
3	The user selects “Sparse Reconstruction-Feature Matching” in the menu bar of the system home page, and the “Feature Matching” dialog box pops up.
4	Depending upon the user requirements and the actual situation in the scene, determine the farthest distance of image-matching pairs, the maximum number of neighbors for each image (the number of pairs to match), and the maximum number of matches between image-matching pairs, as shown in Figure 10b.
5	Upon selecting “Sparse Reconstruction-BA” from the menu bar of the system home page, the “BA” dialog box appears.
6	In Figure 10c, the user specifies the number of BA rounds when adding pictures according to the actual requirements, checks the option to perform a global BA, and enters the number of BA rounds.
7	As shown in Figure 10d, the “Format Conversion” dialog box appears when the user selects “Sparse Reconstruction-Format Conversion” in the menu bar of the system home page.
8	Case 1: Output model format. The user may only select the output sparse point cloud model format. That is, the default is the model format following the 3D reconstruction of the sparse point cloud. Case 2: Convert model format. The user can convert the existing point cloud model to another point cloud format supported by the system.

## Data Availability

The data presented in this study are available on request from the corresponding author.

## References

[1] Schonberger J.L., Frahm J.M. Structure-from-motion revisited. Proceedings of the IEEE Conference on Computer Vision and Pattern Recognition.

[2] Özyeşil O., Voroninski V., Basri R., Singer A. (2017). A survey of structure from motion. Acta Numer..

[3] Iglhaut J., Cabo C., Puliti S., Piermattei L., O’Connor J., Rosette J. (2019). Structure from motion photogrammetry in forestry: A review. Curr. For. Rep..

[4] Agarwal S., Furukawa Y., Snavely N., Simon I., Curless B., Seitz S.M., Szeliski R. (2011). Building rome in a day. Commun. ACM.

[5] Frahm J.M., Fite-Georgel P., Gallup D., Johnson T., Raguram R., Wu C., Jen Y.H., Dunn E., Clipp B., Lazebnik S. Building rome on a cloudless day. Proceedings of the European Conference on Computer Vision.

[6] Jiang N., Tan P., Cheong L.F. Seeing double without confusion: Structure-from-motion in highly ambiguous scenes. Proceedings of the 2012 IEEE Conference on Computer Vision and Pattern Recognition.

[7] Wu C. Towards linear-time incremental structure from motion. Proceedings of the 2013 International Conference on 3D Vision-3DV 2013.

[8] Ni K., Steedly D., Dellaert F. Out-of-core bundle adjustment for large-scale 3d reconstruction. Proceedings of the 2007 IEEE 11th International Conference on Computer Vision.

[9] Triggs B., McLauchlan P.F., Hartley R.I., Fitzgibbon A.W. Bundle adjustment—A modern synthesis. Proceedings of the International Workshop on Vision Algorithms. Springer.

[10] Agarwal S., Snavely N., Seitz S.M., Szeliski R. Bundle adjustment in the large. Proceedings of the European Conference on Computer Vision.

[11] Kneip L., Scaramuzza D., Siegwart R. A novel parametrization of the perspective-three-point problem for a direct computation of absolute camera position and orientation. Proceedings of the CVPR 2011.

[12] Wu C., Agarwal S., Curless B., Seitz S.M. Multicore bundle adjustment. Proceedings of the CVPR 2011.

[13] Eriksson A., Bastian J., Chin T.J., Isaksson M. A consensus-based framework for distributed bundle adjustment. Proceedings of the IEEE Conference on Computer Vision and Pattern Recognition.

[14] Arie-Nachimson M., Kovalsky S.Z., Kemelmacher-Shlizerman I., Singer A., Basri R. Global motion estimation from point matches. Proceedings of the 2012 Second International Conference on 3D Imaging, Modeling, Processing, Visualization & Transmission.

[15] Brand M., Antone M., Teller S. Spectral solution of large-scale extrinsic camera calibration as a graph embedding problem. Proceedings of the European Conference on Computer Vision.

[16] Carlone L., Tron R., Daniilidis K., Dellaert F. Initialization techniques for 3D SLAM: A survey on rotation estimation and its use in pose graph optimization. Proceedings of the 2015 IEEE iNternational Conference on Robotics and Automation.

[17] Chatterjee A., Govindu V.M. Efficient and robust large-scale rotation averaging. Proceedings of the IEEE International Conference on Computer Vision.

[18] Cui Z., Tan P. Global structure-from-motion by similarity averaging. Proceedings of the IEEE International Conference on Computer Vision.

[19] Cui Z., Jiang N., Tang C., Tan P. (2015). Linear global translation estimation with feature tracks. arXiv.

[20] Govindu V.M. Combining two-view constraints for motion estimation. Proceedings of the 2001 IEEE Computer Society Conference on Computer Vision and Pattern Recognition (CVPR 2001).

[21] Govindu V.M. Lie-algebraic averaging for globally consistent motion estimation. Proceedings of the 2004 IEEE Computer Society Conference on Computer Vision and Pattern Recognition (CVPR 2004).

[22] Ozyesil O., Singer A. Robust camera location estimation by convex programming. Proceedings of the IEEE Conference on Computer Vision and Pattern Recognition.

[23] Hartley R., Trumpf J., Dai Y., Li H. (2013). Rotation averaging. Int. J. Comput. Vis..

[24] Sweeney C., Fragoso V., Höllerer T., Turk M. Large scale sfm with the distributed camera model. Proceedings of the 2016 Fourth International Conference on 3D Vision.

[25] Crandall D., Owens A., Snavely N., Huttenlocher D. Discrete-continuous optimization for large-scale structure from motion. Proceedings of the CVPR 2011.

[26] Cui H., Shen S., Gao W., Hu Z. (2015). Efficient large-scale structure from motion by fusing auxiliary imaging information. IEEE Trans. Image Process..

[27] Sweeney C., Sattler T., Hollerer T., Turk M., Pollefeys M. Optimizing the viewing graph for structure-from-motion. Proceedings of the IEEE international Conference on Computer Vision.

[28] Cui H., Gao X., Shen S., Hu Z. HSfM: Hybrid structure-from-motion. Proceedings of the IEEE Conference on Computer Vision and Pattern Recognition.

[29] Zhu S., Shen T., Zhou L., Zhang R., Wang J., Fang T., Quan L. (2017). Parallel structure from motion from local increment to global averaging. arXiv.

[30] Seo Y., Hartley R. A fast method to minimize error norm for geometric vision problems. Proceedings of the 2007 IEEE 11th International Conference on Computer Vision.

[31] Zhu S., Zhang R., Zhou L., Shen T., Fang T., Tan P., Quan L. Very large-scale global sfm by distributed motion averaging. Proceedings of the IEEE Conference on Computer Vision and Pattern Recognition.

[32] Furukawa Y., Hernández C. (2015). Multi-view stereo: A tutorial. Found. Trends Comput. Graph. Vis..

[33] Seitz S.M., Curless B., Diebel J., Scharstein D., Szeliski R. A comparison and evaluation of multi-view stereo reconstruction algorithms. Proceedings of the 2006 IEEE Computer Society Conference on Computer Vision and Pattern Recognition (CVPR 2006).

[34] Goesele M., Curless B., Seitz S.M. Multi-view stereo revisited. Proceedings of the 2006 IEEE Computer Society Conference on Computer Vision and Pattern Recognition (CVPR 2006).

[35] Kar A., Häne C., Malik J. (2017). Learning a multi-view stereo machine. Adv. Neural Inf. Process. Syst..

[36] Vogiatzis G., Hernández C. (2011). Video-based, real-time multi-view stereo. Image Vis. Comput..

[37] Bailer C., Finckh M., Lensch H. Scale robust multi view stereo. Proceedings of the European Conference on Computer Vision.

[38] Hiep V.H., Keriven R., Labatut P., Pons J.P. Towards high-resolution large-scale multi-view stereo. Proceedings of the 2009 IEEE Conference on Computer Vision and Pattern Recognition.

[39] Habbecke M., Kobbelt L. A surface-growing approach to multi-view stereo reconstruction. Proceedings of the 2007 IEEE Conference on Computer Vision and Pattern Recognition.

[40] Goesele M., Snavely N., Curless B., Hoppe H., Seitz S.M. Multi-view stereo for community photo collections. Proceedings of the 2007 IEEE 11th International Conference on Computer Vision.

[41] Furukawa Y., Ponce J. (2009). Accurate, dense, and robust multiview stereopsis. IEEE Trans. Pattern Anal. Mach. Intell..

[42] Seitz S.M., Dyer C.R. (1999). Photorealistic scene reconstruction by voxel coloring. Int. J. Comput. Vis..

[43] Vogiatzis G., Torr P.H., Cipolla R. Multi-view stereo via volumetric graph-cuts. Proceedings of the 2005 IEEE Computer Society Conference on Computer Vision and Pattern Recognition (CVPR 2005).

[44] Sinha S.N., Mordohai P., Pollefeys M. Multi-view stereo via graph cuts on the dual of an adaptive tetrahedral mesh. Proceedings of the 2007 IEEE 11th International Conference on Computer Vision.

[45] Li J., Li E., Chen Y., Xu L., Zhang Y. Bundled depth-map merging for multi-view stereo. Proceedings of the 2010 IEEE Computer Society Conference on Computer Vision and Pattern Recognition.

[46] Shen S. Depth-map merging for multi-view stereo with high resolution images. Proceedings of the 21st International Conference on Pattern Recognition (ICPR 2012).

[47] Shen S., Hu Z. (2013). How to select good neighboring images in depth-map merging based 3D modeling. IEEE Trans. Image Process..

[48] Liu H., Tang X., Shen S. (2020). Depth-map completion for large indoor scene reconstruction. Pattern Recognit..

[49] Wei W., Wei G., ZhanYi H. (2014). Dense 3D scene reconstruction based on semantic constraint and graph cuts. Sci. Sin. Inf..

[50] Schönberger J.L., Zheng E., Frahm J.M., Pollefeys M. Pixelwise view selection for unstructured multi-view stereo. Proceedings of the European Conference on Computer Vision.

[51] Merrell P., Akbarzadeh A., Wang L., Mordohai P., Frahm J.M., Yang R., Nistér D., Pollefeys M. Real-time visibility-based fusion of depth maps. Proceedings of the 2007 IEEE 11th International Conference on Computer Vision.

[52] Yao Y., Luo Z., Li S., Fang T., Quan L. Mvsnet: Depth inference for unstructured multi-view stereo. Proceedings of the European Conference on Computer Vision (ECCV).

[53] Yu Z., Gao S. Fast-mvsnet: Sparse-to-dense multi-view stereo with learned propagation and gauss-newton refinement. Proceedings of the IEEE/CVF Conference on Computer Vision and Pattern Recognition.

[54] Aanæs H., Jensen R.R., Vogiatzis G., Tola E., Dahl A.B. (2016). Large-scale data for multiple-view stereopsis. Int. J. Comput. Vis..

[55] Khot T., Agrawal S., Tulsiani S., Mertz C., Lucey S., Hebert M. (2019). Learning unsupervised multi-view stereopsis via robust photometric consistency. arXiv.

[56] Dai Y., Zhu Z., Rao Z., Li B. Mvs2: Deep unsupervised multi-view stereo with multi-view symmetry. Proceedings of the 2019 International Conference on 3D Vision.

[57] Huang B., Yi H., Huang C., He Y., Liu J., Liu X. M3VSNet: Unsupervised multi-metric multi-view stereo network. Proceedings of the 2021 IEEE International Conference on Image Processing.

[58] Xu H., Zhou Z., Qiao Y., Kang W., Wu Q. Self-supervised multi-view stereo via effective co-segmentation and data-augmentation. Proceedings of the AAAI Conference on Artificial Intelligence.

[59] Garland M., Heckbert P.S. Simplifying surfaces with color and texture using quadric error metrics. Proceedings of the Proceedings Visualization’98 (Cat. No. 98CB36276).

[60] Hoppe H. New quadric metric for simplifying meshes with appearance attributes. Proceedings of the Proceedings Visualization’99 (Cat. No. 99CB37067).

[61] Williams N., Luebke D., Cohen J.D., Kelley M., Schubert B. Perceptually guided simplification of lit, textured meshes. Proceedings of the 2003 Symposium on Interactive 3D Graphics.

[62] Lindstrom P., Turk G. (2000). Image-driven simplification. ACM Trans. Graph..

[63] Li W., Chen Y., Wang Z., Zhao W., Chen L. An improved decimation of triangle meshes based on curvature. Proceedings of the International Conference on Rough Sets and Knowledge Technology.

[64] An G., Watanabe T., Kakimoto M. Mesh simplification using hybrid saliency. Proceedings of the 2016 International Conference on Cyberworlds.

[65] Jiang Y., Nie W., Tang L., Liu Y., Liang R., Hao X. (2016). Vertex Mesh Simplification Algorithm Based on Curvature and Distance Metric. Transactions on Edutainment XII.

[66] TaubinÝ G. Geometric signal processing on polygonal meshes. Proceedings of the Proceedings of Eurographics.

[67] Desbrun M. Processing irregular meshes. Proceedings of the Proceedings SMI, Shape Modeling International 2002.

[68] Fleishman S., Cohen-Or D., Silva C.T. (2005). Robust moving least-squares fitting with sharp features. ACM Trans. Graph..

[69] Bajaj C.L., Xu G. Adaptive fairing of surface meshes by geometric diffusion. Proceedings of the Proceedings Fifth International Conference on Information Visualisation.

[70] Hildebrandt K., Polthier K. (2004). Anisotropic filtering of non-linear surface features. Comput. Graph. Forum.

[71] Lee K.W., Wang W.P. Feature-preserving mesh denoising via bilateral normal filtering. Proceedings of the Ninth International Conference on Computer Aided Design and Computer Graphics (CAD-CG’05).

[72] Wu C. (2011). VisualSFM: A Visual Structure from Motion System. http://ccwu.me/vsfm/index.html.

[73] Furukawa Y. (2010). Clustering Views for Multi-View Stereo. https://www.di.ens.fr/cmvs/.

[74] Fuhrmann S., Langguth F., Goesele M. Mve-a multi-view reconstruction environment. Proceedings of the GCH.

[75] Waechter M., Moehrle N., Goesele M. Let there be color! Large-scale texturing of 3D reconstructions. Proceedings of the European Conference on Computer Vision.

[76] Moulon P., Monasse P., Perrot R., Marlet R. Openmvg: Open multiple view geometry. Proceedings of the International Workshop on Reproducible Research in Pattern Recognition.

[77] Cernea D. (2020). OpenMVS: Multi-View Stereo Reconstruction Library. https://cdcseacave.github.io/openMVS.

[78] Schönberger J.L., Price T., Sattler T., Frahm J.M., Pollefeys M. A Vote-and-Verify Strategy for Fast Spatial Verification in Image Retrieval. Proceedings of the Asian Conference on Computer Vision (ACCV 2016).

[79] Verhoeven G. (2011). Taking computer vision aloft–archaeological three-dimensional reconstructions from aerial photographs with photoscan. Archaeol. Prospect..

[80] Shi J., Malik J. (2000). Normalized cuts and image segmentation. IEEE Trans. Pattern Anal. Mach. Intell..

[81] Martinec D., Pajdla T. Robust rotation and translation estimation in multiview reconstruction. Proceedings of the 2007 IEEE Conference on Computer Vision and Pattern Recognition.

[82] Xu Q., Tao W. Multi-scale geometric consistency guided multi-view stereo. Proceedings of the IEEE/CVF Conference on Computer Vision and Pattern Recognition.

[83] Wang F., Galliani S., Vogel C., Speciale P., Pollefeys M. Patchmatchnet: Learned multi-view patchmatch stereo. Proceedings of the IEEE/CVF Conference on Computer Vision and Pattern Recognition.

[84] Xu Q., Kong W., Tao W., Pollefeys M. (2022). Multi-Scale Geometric Consistency Guided and Planar Prior Assisted Multi-View Stereo. IEEE Trans. Pattern Anal. Mach. Intell..

[85] Golias N., Dutton R. (1997). Delaunay triangulation and 3D adaptive mesh generation. Finite Elem. Anal. Des..

[86] Li Y., Xie Y., Wang X., Luo X., Qi Y. A Fast Method for Large-Scale Scene Data Acquisition and 3D Reconstruction. Proceedings of the 2019 IEEE International Symposium on Mixed and Augmented Reality Adjunct (ISMAR-Adjunct).

[87] Liu J., Ji S. A novel recurrent encoder-decoder structure for large-scale multi-view stereo reconstruction from an open aerial dataset. Proceedings of the IEEE/CVF Conference on Computer Vision and Pattern Recognition.

